# A clinical-radiomics nomogram for the preoperative prediction of lymph node metastasis in colorectal cancer

**DOI:** 10.1186/s12967-020-02215-0

**Published:** 2020-01-30

**Authors:** Menglei Li, Jing Zhang, Yibo Dan, Yefeng Yao, Weixing Dai, Guoxiang Cai, Guang Yang, Tong Tong

**Affiliations:** 1 Department of Radiology, Fudan University Shanghai Cancer Center, Fudan University, Shanghai, 200032 People’s Republic of China; 2 Department of Oncology, Shanghai Medical College, Fudan University, Shanghai, 200032 People’s Republic of China; 3 Shanghai Key Laboratory of Magnetic Resonance, East China Normal University, Shanghai, 200062 People’s Republic of China; 4 Department of Colorectal Surgery, Fudan University Shanghai Cancer Center, Fudan University, Shanghai, 200032 People’s Republic of China

## Abstract

**Background:**

Accurate lymph node metastasis (LNM) prediction in colorectal cancer (CRC) patients is of great significance for treatment decision making and prognostic evaluation. We aimed to develop and validate a clinical-radiomics nomogram for the individual preoperative prediction of LNM in CRC patients.

**Methods:**

We enrolled 766 patients (458 in the training set and 308 in the validation set) with clinicopathologically confirmed CRC. We included nine significant clinical risk factors (age, sex, preoperative carbohydrate antigen 19-9 (CA19-9) level, preoperative carcinoembryonic antigen (CEA) level, tumor size, tumor location, histotype, differentiation and M stage) to build the clinical model. We used analysis of variance (ANOVA), relief and recursive feature elimination (RFE) for feature selection (including clinical risk factors and the imaging features of primary lesions and peripheral lymph nodes), established classification models with logistic regression analysis and selected the respective candidate models by fivefold cross-validation. Then, we combined the clinical risk factors, primary lesion radiomics features and peripheral lymph node radiomics features of the candidate models to establish combined predictive models. Model performance was assessed by the area under the receiver operating characteristic (ROC) curve (AUC). Finally, decision curve analysis (DCA) and a nomogram were used to evaluate the clinical usefulness of the model.

**Results:**

The clinical-primary lesion radiomics-peripheral lymph node radiomics model, with the highest AUC value (0.7606), was regarded as the candidate model and had good discrimination and calibration in both the training and validation sets. DCA demonstrated that the clinical-radiomics nomogram was useful for preoperative prediction in the clinical environment.

**Conclusion:**

The present study proposed a clinical-radiomics nomogram with a combination of clinical risk factors and radiomics features that can potentially be applied in the individualized preoperative prediction of LNM in CRC patients.

## Background

Colorectal cancer (CRC) is the third most common cancer and the fourth leading cause of cancer death worldwide [1]. Lymph node metastasis (LNM) is the main metastatic mode of CRC and an important cause of postoperative recurrence and death [2]. At the same time, the metastasis of lymph nodes (LNs) determines the surgical range of CRC, the formulation of adjuvant treatment plans, and the postoperative survival rate of patients [3–5]. Currently, surgical treatment is the preferred method for the treatment of CRC. However, the operative treatment is relatively invasive, costly, and associated with considerable surgery-related morbidity. The postoperative mortality of colon and rectal cancer surgery has been reported to be approximately 3–6% [6]. For early colorectal cancer (ECC), local treatment such as endoscopic local resection is considered feasible management for patients without LNM because of its low risk of metastasis (3.6–16.2%) [7, 8]. In other words, additional radical resection may be unnecessary for ECC without LNM to avoid overtreatment. It is well known that LN status is a key factor in the TNM staging of CRC. For patients with stage III and IV CRC, modern treatment regimens have a significant impact on long-term survival. Therefore, determining the status of LNs is the main determinant of adjuvant chemotherapy [5, 9]. Multiple large studies have shown that the LNM rate (LNR) is an important predictive factor for estimating the prognosis of CRC [2, 10, 11]. Therefore, adequate and accurate LN status assessment and prediction are important for treatment decision making and prognostic evaluation in CRC.

At present, an increasing number of studies are trying to explore the relevant risk factors for tumorigenesis and metastasis from the perspective of tumor molecular immunology or genetics [12–14]. For instance, Zhang et al. [12] found that IBSP, MMP9, TNFAIP6, DHRS3, RIPK4, and CD200 had diagnostic value for patients with breast cancer bone metastasis. Studies on risk factors associated with CRC are also emerging [7, 8, 15, 16]. For example, the group of Jerome Galon has extensively investigated the important impact of immune-related characteristics and the tumor microenvironment on the prognosis and metastasis of CRC [16]. However, the limitation of using those risk indicators to assess LNM in CRC patients is that some of the influencing factors are related to histopathological information, which cannot be obtained before surgery to guide clinical treatment. In clinical practice, computed tomography (CT) is the most commonly used preoperative imaging method for detecting metastatic lesions and performing tumor staging in patients with colorectal cancer. However, the limitation of CT examination is that it cannot accurately identify the benign and malignant lymph nodes [17]. Hence, we need to develop more powerful and sensitive diagnostic tools to improve the diagnostic accuracy of LNM in CRC patients.

In recent years, with the advancement of computer technology and the realization of the storage and sharing of medical image data, radiomics has become an emerging research method for extracting lesion information using artificial intelligence methods to aid clinical decision making. Radiomics is the process of converting medical images into high-dimensional mineable data through the high-throughput extraction of image features [18, 19]. At present, some studies have demonstrated the feasibility of radiomics for the prediction of CRC LNM. Huang et al. [20] established a radiomics model for predicting CRC LNM (C-index: 0.778) that had favorable discrimination and calibration and incorporated clinicopathological risk factors, the CT-reported LN status and a radiomics signature. However, in their study, only the radiomics features of the primary tumor were extracted and analyzed, and the radiomics features of the LNs themselves were not explored.

Therefore, in this study, we sought to analyze and explore the performance of clinical risk factor and radiomics signatures, including imaging features of the primary lesion and peripheral LNs, in predicting LNM. Then we built and validated a combined clinical-radiomics nomogram as a useful clinical tool for the individualized preoperative prediction of LNM in CRC patients.

## Methods

### Patients

**Fig. 1:**
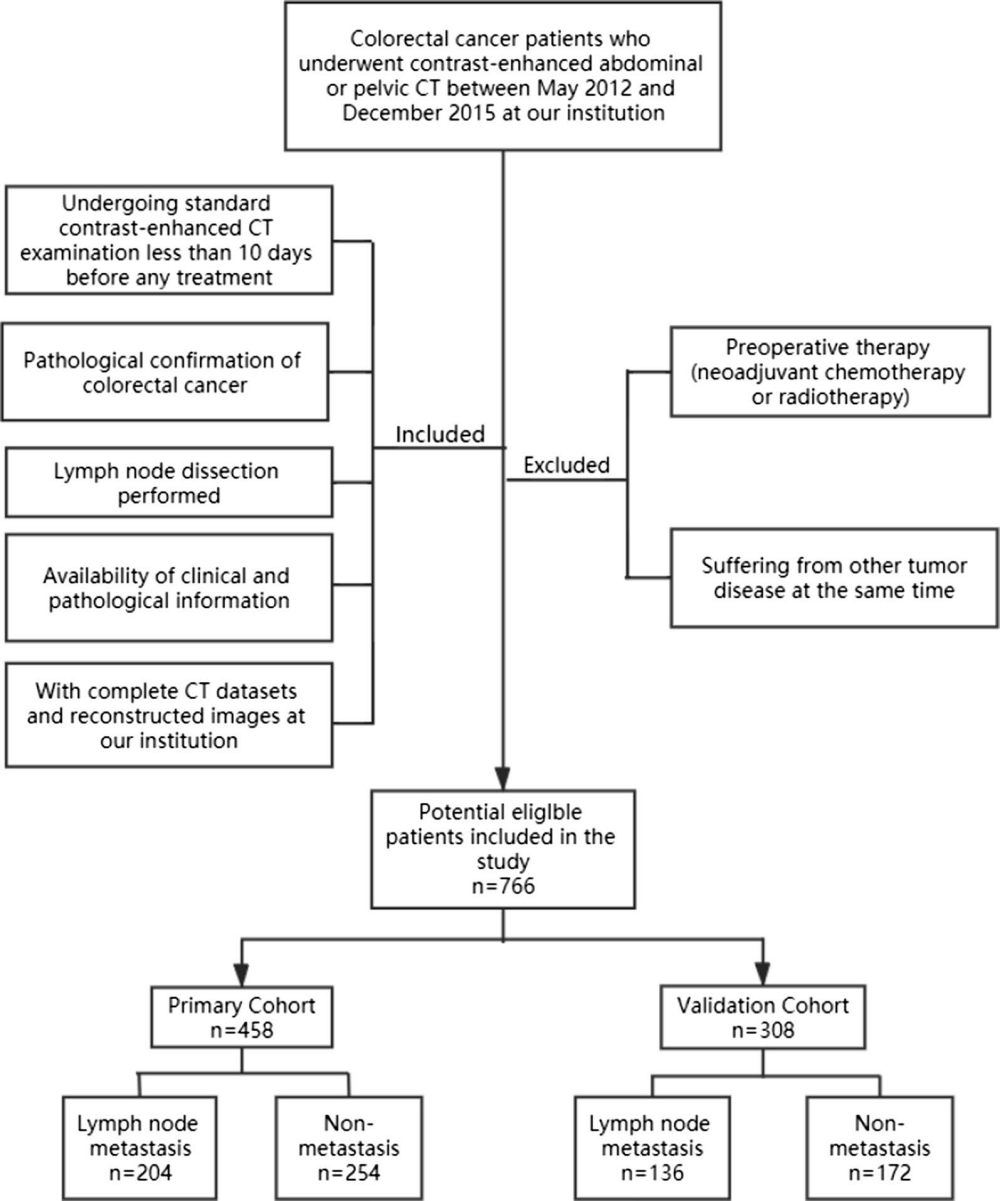
Flow chart of patients’ recruitment pathway

Our Institutional Review Board (Fudan University Shanghai Cancer Center Medical Ethics Committee) approved this study and waived the requirement for obtain informed consent. A total of 766 consecutive CRC patients (341 females and 425 males, mean age 58.96 ± 12.03 years, age range 19–87 years) who were treated between May 2012 and December 2015 were enrolled in our study according to the following inclusion criteria: (i) performance of standard contrast-enhanced CT examination less than 10 days before any treatment; (ii) pathological confirmation of CRC; (iii) performance of LN dissection; (iv) availability of complete CT datasets and reconstructed images; and (v) availability of clinical and pathological information. The exclusion criteria were as follows: (i) preoperative neoadjuvant chemotherapy or radiotherapy; and (ii) presence of other tumor diseases during the same period. The patients were allocated to a training and a validation set at a ratio of 6:4 by the scanning date: the early data before the 60th percentile scanning date were allocated to the training set, and the other data were allocated to the validation set. The patient recruitment pathway is shown in Fig. 1.

The baseline clinical characteristics and pathologic data of each patient, including age, sex, preoperative carbohydrate antigen 19-9 (CA19-9) level, preoperative carcinoembryonic antigen (CEA) level, pathological grading, histotype, tumor location, tumor size and M stage, were all derived from medical records and were preoperatively available. The CT reports of the enrolled patients were also collected.

### Image acquisition and segmentation

All patients underwent contrast-enhanced abdominal or pelvic CT according to standard clinical scanning protocols (120 kV, 200 mA, and slice thickness of 5 mm) with the Brilliance (Philips Healthcare) and the Sensation 64 (Siemens Healthcare) systems. All images were reconstructed with a standard reconstruction kernel as follows: slice thickness, 5.0 mm; pitch, 1.4 or 0.9; increment, 5.0 mm; matrix, 512 × 512; and field of view, 4.11 cm. We retrieved all reconstructed images from the picture archiving and communication system (PACS) in the hospital for image segmentation and analysis.

Three-dimensional semiautomatic segmentation was conducted by an operator (ML, graduate student) with ITK-SNAP software (v3.6.0; www.itksnap.org) and then validated by a senior expert radiologist (TT, with 11 years of experience in CRC). In this study, the regions of interest (ROIs) included the primary lesion and the peripheral LN, which were drawn on the portal venous enhanced CT images along the lesion contour on each consecutive slice within the borders of the primary tumor lesions, excluding adjacent air, vessels, fat and normal tissues.

### Radiomics feature extraction and selection

We extracted radiomics features from each ROI with PyRadiomics (http://pyradiomics.readthedocs.io/en/latest/index.html). Because of some impact on the imaging features, we normalized each CT image by centering it at the mean with standard deviation to eliminate the influence of the different ranges of gray values in PyRadiomics. The classes of the features used included first order, shape, gray level cooccurrence matrix, gray level run length matrix, gray level size zone matrix, gray level dependence matrix and neighboring gray tone difference matrix. Since some of the features were redundant, we used the Pearson correlation coefficient to remove the redundant features. We applied normalization on the feature matrix. Each feature vector was subtracted by the mean value and divided by the standard deviation.

Analysis of variance (ANOVA), Relief and recursive feature elimination (RFE) were used for feature selection. For the radiomics features that remained, we selected the top 20 features from all clinical and radiomics features, and the number of selected features was iterated from 1 to 20. The best feature number was found by comparing the performance of fivefold cross-validation on the training cohort by logistic regression (LR).

### Model construction

The clinical model was constructed by multivariable LR analysis using nine clinical parameters, including age, sex, preoperative CA19-9 level, preoperative CEA level, pathological grading, histotype, tumor location, tumor size and M stage. Akaike’s information criterion was used as the stopping rule for backward step wise selection. Then, a multivariate LR model was established and its diagnostic performance was tested on the training cohort.

Two radiomics models were constructed with the selected radiomics features from the primary lesion and the peripheral LN ROI respectively.

We also constructed three clinical-radiomics models combining the radiomics and clinical features with a LR mode and evaluated the performance of the combined models.

Hence, a total of six models were constructed to preoperatively predict the LNM of CRC: 1 clinical-only model, 2 radiomics-only models (the LN radiomics model and the lesion radiomics model) and 3 combined clinical-radiomics models (clinical-lesion, clinical-LN and clinical-lesion-LN).

### Model validation and comparison

Receiver operating characteristic (ROC) curve analysis was used to assess the model performance in the training and testing cohorts. The area under the ROC curve (AUC) was calculated for quantification. We regarded the model with the highest AUC value in the fivefold cross-validation of the training set as the candidate model. The accuracy, sensitivity, specificity, positive predictive value (PPV) and negative predictive value (NPV) were also calculated at a cutoff value that maximized the value of the Youden index. Moreover, we also boosted the estimation 1000 times and applied paired t-test to determine the 95% confidence intervals (CIs).

### Nomogram development and decision curve analysis (DCA)

A nomogram was generated for model visualization and clinical application. To evaluate the added value of radiomics features to clinical features in individually predicting LNM in CRC patients, we developed six decision curves based on the clinical parameters, primary lesion radiomics features, LN radiomics features and the combined clinical-radiomics models. The clinical utility could be demonstrated by quantifying the net benefits of a series of threshold probabilities in the queue.

## Results

### Clinical features

The LNM rates of the training set and the validation set were 44.15% and 44.54%, respectively. There were no significant differences in patient age, sex, preoperative CEA level, preoperative CA19-9 level, pathological grading, histotype, tumor location, tumor size and M stage between the training set and the validation set (P > 0.05), as shown in Table 1.

**Table 1:** Demographic comparison between training and validation cohorts

Characteristic	Training cohort	Validation cohort	P
Age, mean SD	58.86 ± 12.38	59.12 ± 11.53	0.48
Tumor size, mean SD	4.48 ± 1.91	4.53 ± 1.82	0.25
Sex (%)			0.62
Male	258.0 (60.71%)	167.0 (39.29%)	
Female	200.0 (58.65%)	141.0 (41.35%)	
M stage (%)			0.56
M0	399.0 (60.27%)	263.0 (39.73%)	
M1	59.0 (56.73%)	45.0 (43.27%)	
CA19-9 (%)			0.94
0–27 U/mL	362.0 (59.64%)	245.0 (40.36%)	
≥27 U/mL	96.0 (60.38%)	63.0 (39.62%)	
CEA (%)			0.08
0–5 ng/mL	298.0 (62.34%)	180.0 (37.66%)	
≥ 5 ng/mL	160.0 (55.56%)	128.0 (44.44%)	
Location (%)			0.42
Right	208.0 (61.54%)	130.0 (38.46%)	
Left	250.0 (58.41%)	178.0 (41.59%)	
Grade (%)			0.63
High	26.0 (5.68%)	21.0 (6.82%)	
Middle	301.0 (65.72%)	207.0 (67.21%)	
Low	131.0 (28.60%)	80.0 (25.97%)	
Histotype (%)			0.93
Adenocarcinoma	379.0 (82.75%)	258.0 (83.77%)	
Mucinous histology	74.0 (16.16%)	47.0 (15.26%)	
Signet ring cell differentiation	5.0 (1.09%)	3.0 (0.97%)	

Chi-Square or Fisher Exact tests, as appropriate, were used to compare the differences in categorical variables (gender, M stage, CA19-9 level, CEA level, location, grade, histotype), while a two-sample t-test was used to compare the differences in age and tumor size. Laboratory analysis of CEA and CA 19-9 were done via routine blood tests within 1 week before surgery. The threshold value for CEA level was ≤ 5 ng/mL and > 5 ng/mL and the threshold value for CA 19-9 level was ≤ 27 U/mL and > 27 U/mL, according to the normal range used in clinics

*CEA* carcinoembryonic antigen, *CA19-9* carbohydrate antigen 19-9

After multivariate LR analysis, age, sex, pathological grading, histotype, preoperative CA19-9 level and preoperative CEA level were independent predictors in the clinical model.

### Feature extraction and model construction

**Fig. 2:**
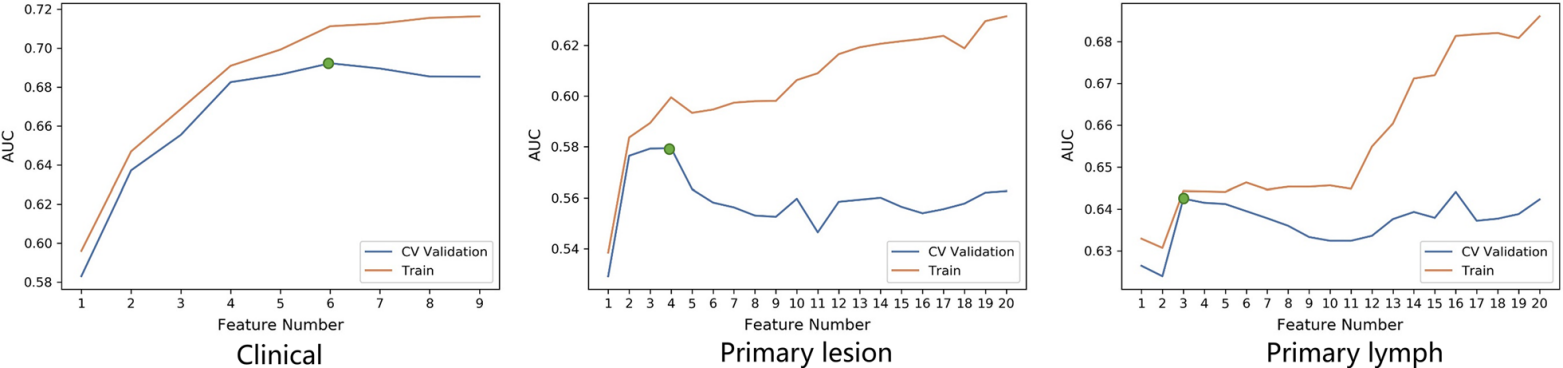
Feature selection. Selection of the tuning feature number in LR model via fivefold cross validation based on minimum criteria. The y-axis indicates AUC value. The x-axis indicates the feature number. The green point indicates model with highest AUC value in fivefold cross validation

A total of 222 radiomics features were extracted from CT images (111 lesion radiomics features and 111 peripheral LN radiomics features). ANOVA, RFE and Relief were used to reduce the feature number to 20. Then we evaluated the candidate feature number in the validation cohort by assessing the discrimination performance. The feature number was validated by fivefold cross-validation. We obtained an candidate subset of 6 clinical features (age, M stage, preoperative CA19-9 level, preoperative CEA level, pathological grading and tumor size), 3 primary lesion radiomics features (Lesion_glcm_ldmn, Lesion_glszm_ZonePercentage, and Lesion_glszm_LowGrayLevelZoneEmphases) and 3 peripheral LN radiomics features (Lymph_glrlm_GrayLevelNonUniformity, and Lymph_firstorder_Kurtosis, Lymph_gldm_GrayLevelNonUniformity) (Fig. 2). Then, we established six predictive models using the above selected features: 1 clinical model, 2 radiomics models and 3 combined clinical-radiomics models.

### Model comparison and validation

**Fig. 3:**
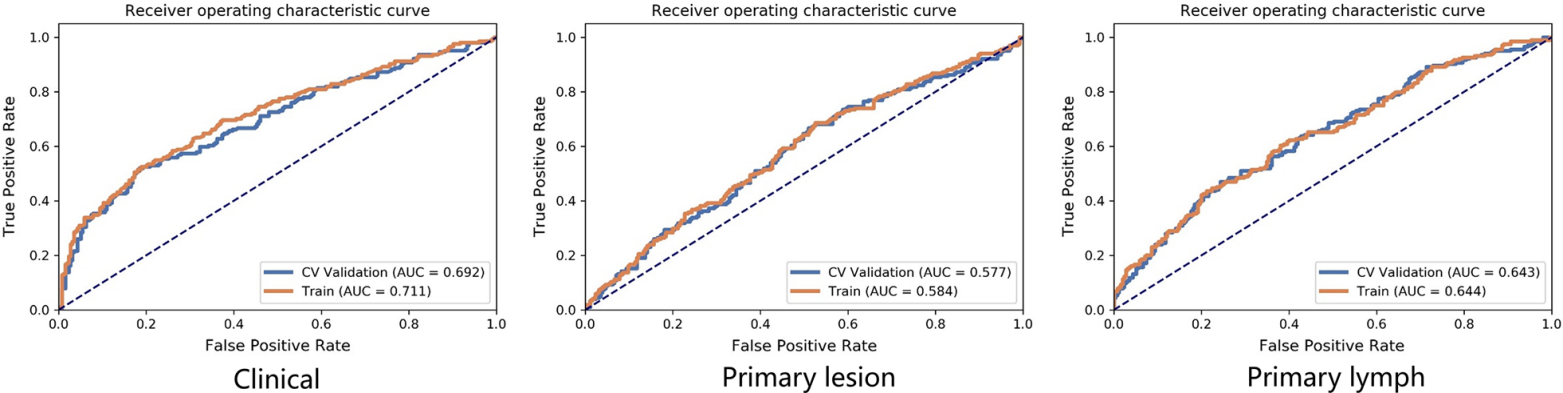
The ROC curves of the clinical features, peripheral lymph node radiomics features and primary lesion radiomics features in the training and validation sets, respectively

**Fig. 4:**
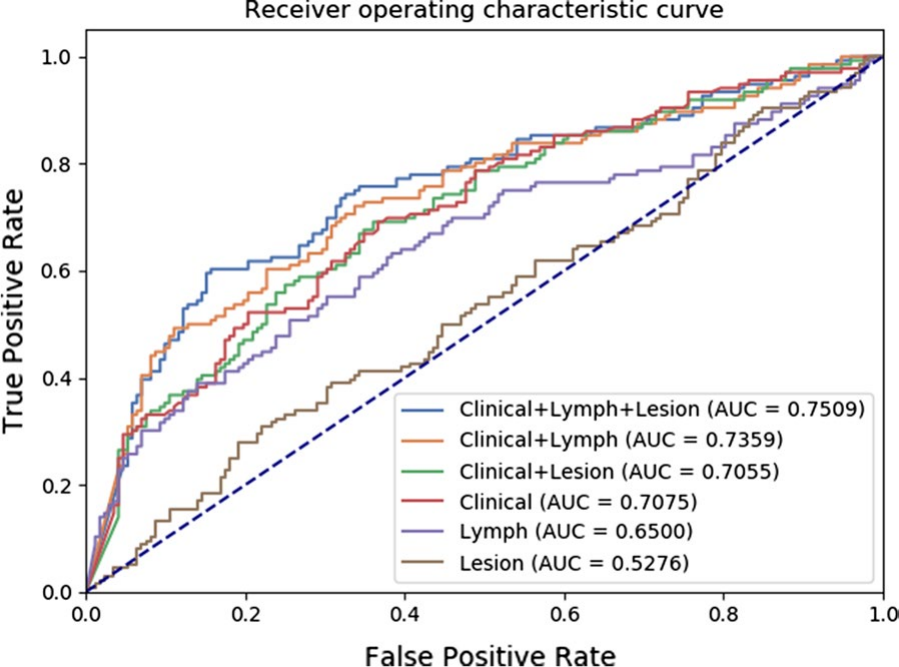
Model performance in validation cohort. Compared with different features combination, the AUC value is increased when clinical parameters joined either lymph feature or lesion feature. We can also see that the more feature species used, the higher AUC value and the better model performance are

Figure 3 shows the performance of the clinical features, peripheral LN radiomics features and primary lesion radiomics features in the training and validation sets, respectively. By comparing the models, the clinical-lesion-LN model presented the optimal discrimination and best predictive stability with the highest AUC value in both the training cohort (AUC = 0.7606; 95% CI 0.7373–0.7833) and the validation cohort (AUC = 0.7509; 95% CI 0.6901–0.8071) (Table 2, Fig. 4). We also compared different parameters of the LR model for better performance, and there was limited influence on the AUC values in the testing set (Additional file 1: Table S1), therefore, we used the default values. Though the combined model seemed complex, both the lesion radiomics features and the LN radiomics features were extracted from the same CT images, so the combined model with great performance does not bring about an extra burden to clinical examinations. Hence, the clinical-lesion-LN model was identified as the candidate classifier model for LNM in CRC patients.

**Table 2:** Accuracy and predictive value between six models

Training cohort	AUC	95% CI	Sensitivity	Specificity	Accuracy	PPV	NPV
Clinical features	0.7127	[0.6876–0.7367]	0.5466	0.7844	0.6785	0.6707	0.6829
Lesion radiomics	0.6007	[0.5756–0.6273]	0.6042	0.5588	0.5790	0.5228	0.6383
Lymph node radiomics	0.6441	[0.6171–0.6681]	0.4363	0.7853	0.6302	0.6106	0.6564
Clinical-lesion radiomics	0.7299	[0.7063–0.7524]	0.5931	0.7951	0.7053	0.6984	0.7095
Clinical-lymph node radiomics	0.7519	[0.7288–0.7738]	0.5637	0.8255	0.7092	0.6282	0.7500
Clinical-lesion-lymph node radiomics	0.7606	[0.7373–0.7833]	0.6495	0.7902	0.7277	0.7124	0.7381

Validation cohort	AUC	95% CI	Sensitivity	Specificity	Accuracy	PPV	NPV
Clinical features	0.7075	[0.6448–0.7650]	0.6912	0.6337	0.6591	0.5987	0.7219
Lesion radiomics	0.5276	[0.4634–0.5927]	0.3088	0.7791	0.5714	0.5250	0.5877
Lymph node radiomics	0.6500	[0.5829–0.7133]	0.5074	0.7442	0.6396	0.6106	0.6564
Clinical-lesion radiomics	0.7055	[0.6459–0.7637]	0.6912	0.6395	0.6623	0.6026	0.7237
Clinical-lymph node radiomics	0.7359	[0.6750–0.7899]	0.7206	0.6628	0.6883	0.6282	0.7500
Clinical-lesion-lymph node radiomics	0.7509	[0.6901–0.8071]	0.6029	0.8430	0.7370	0.7523	0.7286

*AUC* area under the curve, *PPV* positive predictive value, *NPV* negative predictive value

### Clinical use

**Fig. 5:**
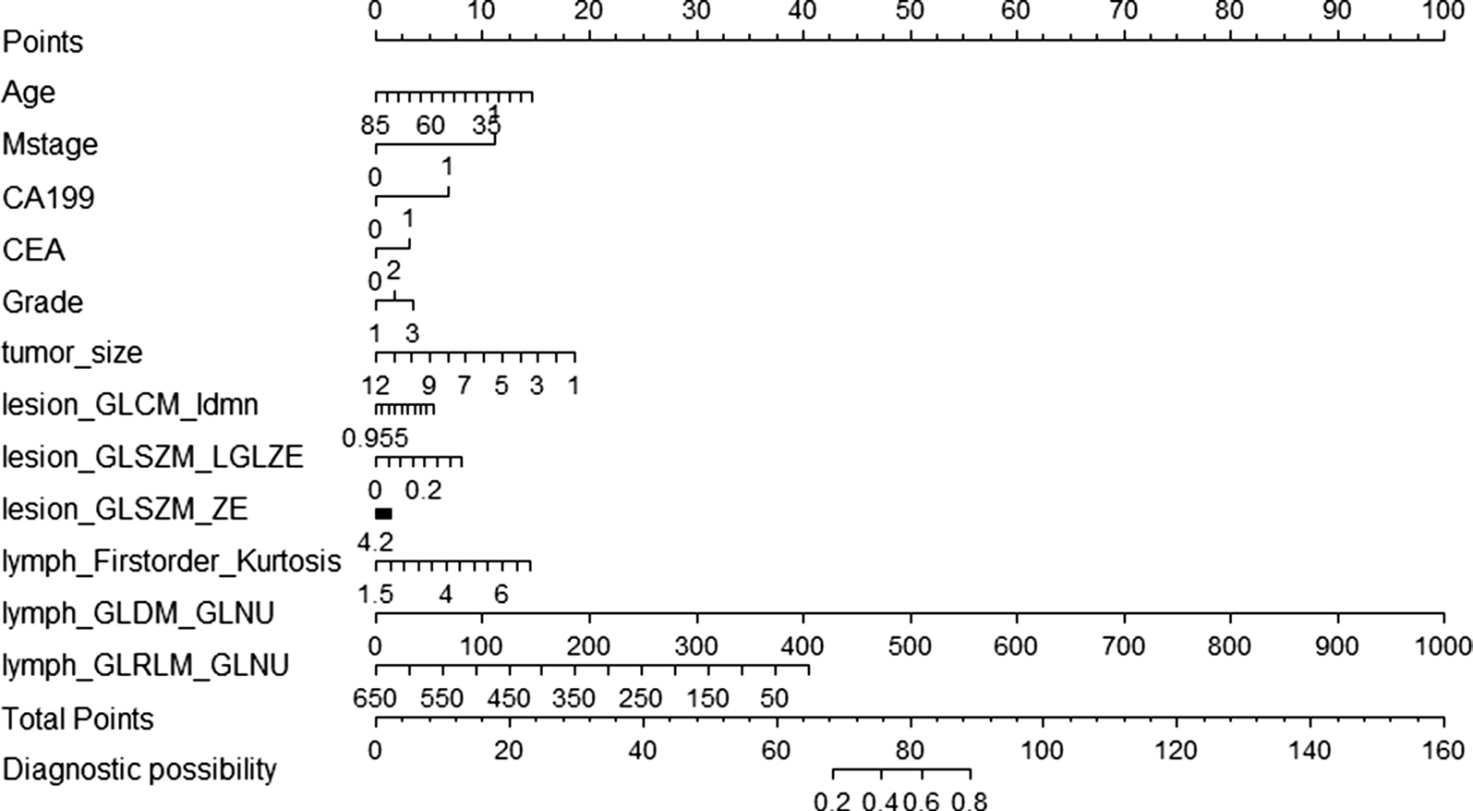
The developed clinical-primary lesion radiomics-peripheral lymph node radiomics nomogram for predicting the probability of lymph node metastases. For age, the number represents the age number. For M stage, 0 represents no distant metastasis while 1 represents distant metastasis. For the preCA19-9, 0 represents within the normal value while 1 represents above the normal value. For the preCEA, 0 represents within the normal value while 1 represents above the normal value. For the grade, 1 for high-middle differentiation and 0 for middle-low differentiation. To use, locate the patient’s age, draw a line straight up to the points axis to establish the score associated with that site. Repeat for the other covariates (M stage, preCA19-9, preCEA, grade and radiomics features). By summing the scores of each point and locating on the total score scale, the estimated probability of lymph node metastases could be determined

**Fig. 6:**
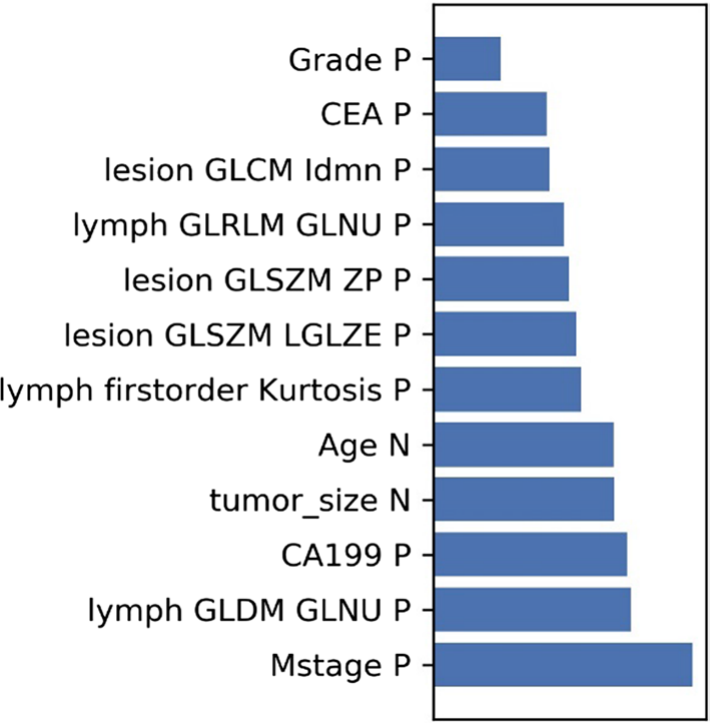
Weight of feature coefficients of the candidate model. Feature weights generated by the coefficients of logistic regression model. The “P” after the feature name indicates the positive correlation, and the “N” indicates the negative correlation. *lesion GLCM IDMN* lesion_CT_original_glcm_Idmn, *lesion GLCM ZP* lesion_CT_original_glcm_ ZonePercentage, *lesion GLSZM LGLZE* lesion_glszm_LowGrayLevelZoneEmphaseslymph, *lymph GLDM GLNU* lymph_glrlm_GrayLevelNonUniformity

Based on this candidate model, we generated a clinical-lesion radiomics-LN radiomics nomogram for model visualization (Fig. 5). We found that the clinical features have higher classification contributions than the radiomics features in the candidate nomogram. This finding is consistent with the higher AUC value of the clinical model than that of the LN radiomics model and the lesion radiomics model. However, the LN radiomics features greatly help in the decision -making of the candidate model and increases the AUC value from 0.7075 to 0.7509. (Table 2, Fig. 6).

**Fig. 7:**
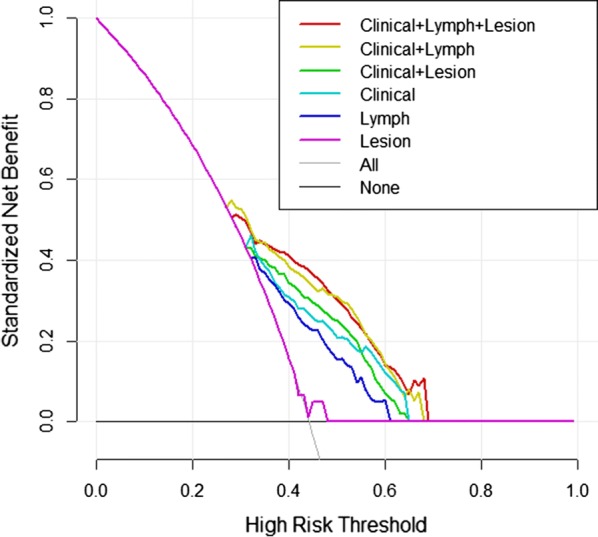
Decision curve analysis (DCAs). The y-axis measures the net benefit. Threshold probability refers to the point at which a patient considers the benefit of treatment for intermediate to high-risk LNM equivalent to the harm of over-treatment for low-risk disease and thus reflects how the patient weights the benefits and harms associated with the decision. The higher curve at any given threshold probability is the optimal prediction to maximize net benefit. The decision curve showed that the model using all feature sets adds more net benefit than other models

DCAs based on the six models are shown in Fig. 7. Both of the single radiomics DCAs showed less benefit in predicting the risk of LNM than the single clinical DCA, which indicates that the clinical features outperformed the radiomics features with more accuracy in LNM prediction. With the addition of radiomic features, the combined clinical-radiomics DCAs achieved more clinical utility, especially the clinical-lesion-LN DCA, which indicated that the nomogram based on the candidate model was a reliable clinical treatment tool for predicting LNM in CRC patients. DCA indicated that when the threshold probability for a patient is within a range from approximately 0.3 to 0.7, the clinical-radiomics nomogram adds more net benefit than the “treat all” or “treat none” strategies.

## Discussion

In this study, we built a clinical-radiomics model for the individualized preoperative prediction of LNM in CRC patients, that consists of clinical risk factors and radiomics features (including imaging features of both the primary lesion and peripheral LNs). First, by multivariate LR analysis, we selected 6 clinical risk factors, 3 primary lesion radiomics features and 3 peripheral LN radiomics features as independent risk factors from 9 clinical features, 111 peripheral LN radiomics features and 111 primary lesion radiomics features, respectively. Then, by using the above independent risk factors, we constructed six predictive models. By comparing the models, the clinical-lesion radiomics-LN radiomics model had the highest accuracy in predicting LNM (AUC = 0.7509; 95% CI 0.6901–0.8071; accuracy: 73.7%; sensitivity: 60.29%; specificity: 84.3%: PPV: 75.23%; and NPV: 72.86% in the testing set) and was regarded as the candidate model. Finally, a nomogram was constructed based on the candidate combined model to achieve model visualization and provide clinical utility. Our study demonstrates that this combined clinical-radiomics model has potential as a clinical tool for preoperatively predicting LNM in CRC patients.

In terms of clinical features, our study found that age, M stage, preoperative CA19-9 level, preoperative CEA level, tumor size and pathological grading were independent risk factors associated with LNM in CRC patients, which is consistent with the findings of previous studies [8, 20–22]. Among the five potential clinical risk factors, the preoperative CA19-9 level and preoperative CEA level have been considered clinical indicators closely related to the LNM of CRC [20]. Our study also suggested that high preoperative CA19-9 levels and high preoperative CEA levels were important predictors of LNM in CRC patients. This finding might be due to the higher level of CEA and CA19-9, the higher TNM stage of CRC and the stronger tumor cell proliferation ability, which implies poorer differentiation, increased invasiveness and stronger metastasis of the tumor [23]. In addition, young patients with CRC were more likely to have LNM, which might be related to the high metabolism of young patients and the occurrence of mostly poorly differentiated tumors. However, most young people do not have obvious symptoms or are not actively undergoing physical examinations, leading to a low clinical detection rate of CRC in this population. In addition, most of them are generally in advanced stages and have a poor prognosis. Therefore, young people should be encouraged to actively undergo early screening so that early diagnosis and early treatment can be performed. There is no consensus on the correlation between tumor size and LNM which might be related to differences in the number of clinical case samples and methods [15, 24, 25]. However, Wolmark et al. [24] also indicated that the tumor volume of Dukes C phase’s rectal cancer was consistently smaller than that of Dukes B tumor. Similarly, we observed a negative correlation between tumor size and LNM in this study. A recent propensity score matching study indicated that a smaller tumor size was an independent risk factor for cancer-specific survival (CSS) in patients with stage I-III CRC [26]. Hence, clinically, multivariate analysis should be used to select the best clinical treatment method to reduce the residual lesion and micro-metastases, thereby reducing the possibility of tumor recurrence and improving the patient’s prognosis and tumor-free survival.

In addition to assessing clinical risk factors, we also tried to explore and find additional imaging information on contrast-enhanced CT images. Compared with traditional imaging methods that can be analyzed from only the level of anatomical changes, the advantage of radiomics is that high-throughput calculations can extract large numbers of quantitative features from the ROI that reflect the inherent heterogeneity of the lesion, which has been widely used in clinical diagnosis, efficacy evaluations, prognostic evaluations and other aspects [27–29]. In 2016, Huang et al. [20] proposed a predictive model of LNM in CRC patients that combined radiomics and clinical indicators (CI = 0.736). However, they analyzed only the radiomics features of the primary lesion. The innovation of our study lies in that we not only analyzed the radiomics features of the primary lesion, but also explored the radiomics features of the peripheral LN themselves. In our study, 3 peripheral LN radiomics features were selected from the 111 screened peripheral LN radiomics features, and these features helped greatly improve the AUC of the final candidate model. This finding indicated that the radiomics features of the LNs themselves might be of greater value in predicting LNM and worthy of further exploration.

Since the combined clinical-lesion-LN radiomics model, with the highest AUC and greatest net benefits across most of the threshold probabilities in DCA, outperformed the single clinical features, single radiomics features and other combined models, it may be the most promising approach to guide clinical management. To facilitate clinical applications, we constructed a clinical-radiomics nomogram combining the radiomics features with preoperative clinical characteristics. The scoring system can generate the probability of LNM to realize the individualized preoperative prediction of the risk of LNM in CRC by clinicians, which is in line with the current development trend of individualized precision medicine.

In conclusion, we recommend that patients with a younger age, distant metastasis, a lower tumor grading, a smaller tumor size and higher preoperative CA19-9 and CEA levels should have regular follow-ups, and the progression of the disease should be closely monitored in these patients. In addition, we suggest that patients with a higher risk of LNM, as screened by the nomogram, should be considered potential surgical candidates to extend survival. The clinical application of this nomogram can reduce the cost of subsequent diagnosis, help develop more reasonable and effective treatment plans and prevent patients from having a poor prognosis.

However, the present study has several limitations. First, as a retrospective study, there may be inevitable selection bias, hence, prospective and external validation studies are needed. Second, the result of this study was from a single institution, so multicenter validation is needed to extend the versatility of the experimental results. Third, only one imaging modality was used in this study, which leads to a limited number of extracted radiomics features. If more imaging modalities (such as MRI and PET-CT) are combined, the feature pool will be effectively expanded to obtain more valuable radiomics information.

## Conclusion

In summary, our study established a clinical-radiomics nomogram that combined clinical risk factors and radiomics features (including radiomics features of the primary lesion and peripheral LNs), which can be used as an individualized preoperative noninvasive tool for predicting LNM in patients with CRC, assisting in clinical treatment decision making and achieving precision treatment.

## Supplementary information

**Additional file 1.** Additional material about the additional descriptions of the clinical features, model building and statistical for software and Table S1 (Performance of different parameters of model).

## Data Availability

All data generated or analysed during this study are included in this published article.

## Abbreviations

LNM: lymph node metastasis
CRC: colorectal cancer
CA19-9: carbohydrate antigen 19-9
CEA: carcinoembryonic antigen
ANOVA: analysis of variance
RFE: recursive feature elimination
ROC: receiver operating characteristic curve
AUC: area under the curve
DCAs: decision curve analysis
ECC: early colorectal cancer
LNR: lymph node metastasis rate
LN: lymph node
CT: computed tomography
PACS: the picture archiving and communication system
ROI: regions of interest
LR: logistic regression
PPV: positive predictive value
NPV: negative predictive value
CIs: confidence intervals
DCA: decision curve analyses
lesion GLCM IDMN: lesion_CT_original_glcm_Idmn
lesion GLCM ZP: lesion_CT_original_glcm_ ZonePercentage
lesion GLSZM LGLZE: lesion_glszm_LowGrayLevelZoneEmphaseslymph
lymph GLDM GLNU: lymph_glrlm_GrayLevelNonUniformity
