# Supplementary material for: A clinical-radiomics nomogram for the preoperative prediction of lymph node metastasis in colorectal cancer
Source: J Transl Med. 2020 Jan 30;18:46. doi: 10.1186/s12967-020-02215-0 (PMC6993349; doi:10.1186/s12967-020-02215-0)
Supplement: Supplementary file 1 — Additional file 1. Additional material about the additional descriptions of the clinical features, model building and statistical for software and Table S1 (Performance of different parameters of model). [file 12967_2020_2215_MOESM1_ESM.docx]

**A clinical-radiomics nomogram for the preoperative prediction of lymph node metastasis in colorectal cancer**

**Additional Material**

1. **Additional Descriptions**

Clinical features: (a) the patients’ gender: male or female; (b) the patients’ age when the lesion of colorectal cancer was firstly detected by CT examination; (c) the primary tumor location: the left colon or the right colon; (d) grade: high-middle differentiation or middle-low differentiation (e) histotype: 1: adenocarcinoma; 2: mucinous adenocarcinoma; 3: others; (f) preCEA level ( tested before any treatment after the lesion of colorectal cancer was firstly detected): with 0～5 ng/ml for normal and ≥5ng/ml for abnormal; (g) preCA19-9 level ( tested before any treatment after the lesion of colorectal cancer was firstly detected): with 0～27 U/ml for normal and ≥27 U/ml for abnormal; (h) tumor size: the primary tumor size; (i) M stage: 0: no distant metastasis; 1: distant metastasis.

1. **Additional Methods**
   1. **Model building and Statistical for software**

All model building process and statistical tests were performed using Python (v 3.7.1). We used sklearn (v 0.20.1) package for feature selection and classification model building, and visualized the result include ROC curve by matplotlib (v 3.0.2). Scipy (v 1.1.0) was used for statistical test, the two-sided p value < 0.05 was considered significant.

We used R software (v 3.5.3) for special visualization. Nomogram construction was performed by using the rms package (v 5.1.3.1). DCA was performed by using the rmda package (v 1.6).

1. **Additional Tables**

**Table S1: Performance of different parameters of model**

| Param | Train AUC | | Val AUC |
| --- | --- | --- | --- |
| C_0.01-max_iter_100-tol_3e-05 | 0.7616 | 0.7417 | |
| C_0.01-max_iter_100-tol_0.0003 | 0.7616 | 0.7418 | |
| C_0.01-max_iter_100-tol_0.003 | 0.7616 | 0.7416 | |
| C_0.01-max_iter_200-tol_3e-05 | 0.7617 | 0.7416 | |
| C_0.01-max_iter_200-tol_0.0003 | 0.7618 | 0.7416 | |
| C_0.01-max_iter_200-tol_0.003 | 0.7618 | 0.7414 | |
| C_0.1-max_iter_100-tol_3e-05 | 0.7618 | 0.7415 | |
| C_0.1-max_iter_100-tol_0.0003 | 0.7618 | 0.7415 | |
| C_0.1-max_iter_100-tol_0.003 | 0.7617 | 0.7415 | |
| C_0.1-max_iter_200-tol_3e-05 | 0.7618 | 0.7415 | |
| C_0.1-max_iter_200-tol_0.0003 | 0.7618 | 0.7415 | |
| C_0.1-max_iter_200-tol_0.003 | 0.7618 | 0.7414 | |
| C_1.0-max_iter_100-tol_3e-05 | 0.7618 | 0.7414 | |
| C_1.0-max_iter_100-tol_0.0003 | 0.7618 | 0.7415 | |
| C_1.0-max_iter_100-tol_0.003 | 0.7618 | 0.7414 | |
| C_1.0-max_iter_200-tol_3e-05 | 0.7618 | 0.7415 | |
| C_1.0-max_iter_200-tol_0.0003 | 0.7618 | 0.7415 | |
| C_1.0-max_iter_200-tol_0.003 | 0.7618 | 0.7414 | |
| C_5-max_iter_100-tol_3e-05 | 0.7618 | 0.7414 | |
| C_5-max_iter_100-tol_0.0003 | 0.7618 | 0.7415 | |
| C_5-max_iter_100-tol_0.003 | 0.7618 | 0.7414 | |
| C_5-max_iter_200-tol_3e-05 | 0.7618 | 0.7414 | |
| C_5-max_iter_200-tol_0.0003 | 0.7618 | 0.7415 | |
| C_5-max_iter_200-tol_0.003 | 0.7618 | 0.7414 | |

Note:

Logistic Regression of scikit-learn uses following parameters:

- C, inverse of regularization strength;
- max_iter, the maximum number of iterations taken for the solvers to converge;
- tol, the tolerance for stopping criteria.

default values of C = 1.0, max_iter = 100, tol = 0.0001
